# Picosecond metrology of laser-driven proton bursts

**DOI:** 10.1038/ncomms10642

**Published:** 2016-02-10

**Authors:** B. Dromey, M. Coughlan, L. Senje, M. Taylor, S. Kuschel, B. Villagomez-Bernabe, R. Stefanuik, G. Nersisyan, L. Stella, J. Kohanoff, M. Borghesi, F. Currell, D. Riley, D. Jung, C.-G. Wahlström, C.L.S. Lewis, M. Zepf

**Affiliations:** 1 Centre for Plasma Physics, School of Mathematics and Physics, Queens University Belfast, Belfast BT7 1NN, UK; 2 Department of Physics, Lund University, PO Box 118, S-221 00 Lund, Sweden; 3 Helmholtz-Institut Jena, Frobelstieg 3, 07743 Jena, Germany; 4 School of Physics, University College Dublin, Belfield, Dublin 4, Ireland; 5 Atomistic Simulation Centre, School of Mathematics and Physics, Queens University Belfast, Belfast BT7 1NN, UK

## Abstract

Tracking primary radiation-induced processes in matter requires ultrafast sources and high precision timing. While compact laser-driven ion accelerators are seeding the development of novel high instantaneous flux applications, combining the ultrashort ion and laser pulse durations with their inherent synchronicity to trace the real-time evolution of initial damage events has yet to be realized. Here we report on the absolute measurement of proton bursts as short as 3.5±0.7 ps from laser solid target interactions for this purpose. Our results verify that laser-driven ion acceleration can deliver interaction times over a factor of hundred shorter than those of state-of-the-art accelerators optimized for high instantaneous flux. Furthermore, these observations draw ion interaction physics into the field of ultrafast science, opening the opportunity for quantitative comparison with both numerical modelling and the adjacent fields of ultrafast electron and photon interactions in matter.

## Abstract (web summary)

Experimental investigations of the response of matter to ionization would require extremely fast ion pump pulses. Here, the authors explore a different approach observing ionisation dynamics in SiO_2_ glass by generating synchronized proton pulses from the interaction of high-power lasers on a solid target.

## Graphical abstract

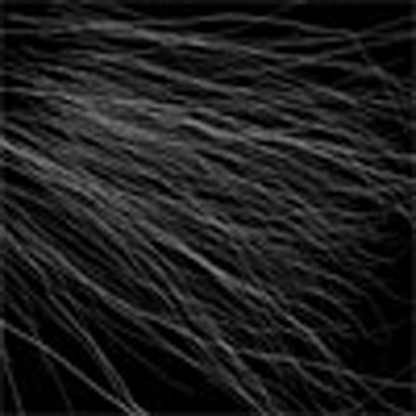

To date the experimental investigation of exactly how matter recovers in response to ion damage, and the emerging pathways for the resultant reactive species generated during the interaction, has been limited by pulse length in conventional accelerators and probe timing jitter^1^. When a pulse of ions (with kinetic energy >1 MeV per nucleon) interacts with condensed matter the individual particles generate nanometre-wide tracks of ionization with correspondingly steep energy density gradients^2^,^3^,^4^,^5^,^6^. The generation of steep energy density gradients drives a rapid diffusion of the hot electron population that tends to homogenize the dose distribution^3^,^4^. This process leads to secondary ionization cascades and the formation of long-lived excited states/chemically reactive species, which equilibrate with the background material over picosecond timescales^7^,^8^. Studying the lifetime of these states is critical for understanding the relationship between the incident ion flux and time-dependent defect concentration in ion-irradiated matter^9^ and cell death/repair rate for radiobiology driven by ultrafast energy deposition^10^. This interaction, and subsequent evolution of dose, is unambiguously distinct from the near-homogeneous excitation that results from both X-ray and electron interactions due to significantly different stopping powers of the different ionizing species in matter.

While pump–probe measurements can in principle reveal these inherently multi-scale processes, they require that the ion pump pulse is significantly shorter than the mean lifetime of the species under investigation and that there is a comparatively high degree of synchronicity between the pump and probe sources. As mentioned above, the corresponding few picosecond bunch duration is not routinely available from conventional accelerators^1^,^11^ and so an alternative source is required. One method to overcome this problem is to capitalize on the ultrafast acceleration phase provided during the interaction of high-power lasers with thin, solid-density foils^12^,^13^,^14^. The high instantaneous flux is suitable for a host of high energy density applications^15^,^16^,^17^,^18^ but to date the excellent synchronicity between the pulse of protons (pump) and the driving laser (probe) has not been exploited to directly investigate ultrafast damage processes in matter^8^,^10^,^19^.

Here we demonstrate an absolute duration measurement of proton pulses generated during high-power laser solid target interactions that overcomes these limitations. Our technique relies on the observation of prompt ionization dynamics^8^,^20^ in high-purity SiO_2_ glass (a transparent wide bandgap dielectric) irradiated by laser-driven protons using highly synchronized optical laser pulses. As part of this measurement we are also able to place an upper limit on the mean free lifetime of the excited electron population in the conduction band of <0.2 ps for our experimental conditions. It is this ultrafast response of SiO_2_ that permits the single-shot temporal characterization (with <0.5 ps resolution for our entire detection system) of the proton pulse driving the ionization dynamics. In addition we demonstrate the general applicability of this technique by extending our measurements to perform jitter-free tracking of ion-induced dynamics in an alternative sample dielectric sample (borosilicate glass). This is made possible because the absolute proton pulse duration has been established. In future this technique can be combined with equally synchronized laser-driven coherent ultraviolet/X-ray probes^21^,^22^ to allow for metrology of ion interactions on timescales of the rising edge of the interaction and potentially detailed studies of the initial damage track structure in the medium.

## Results

### Experimental procedure

**Figure 1:**
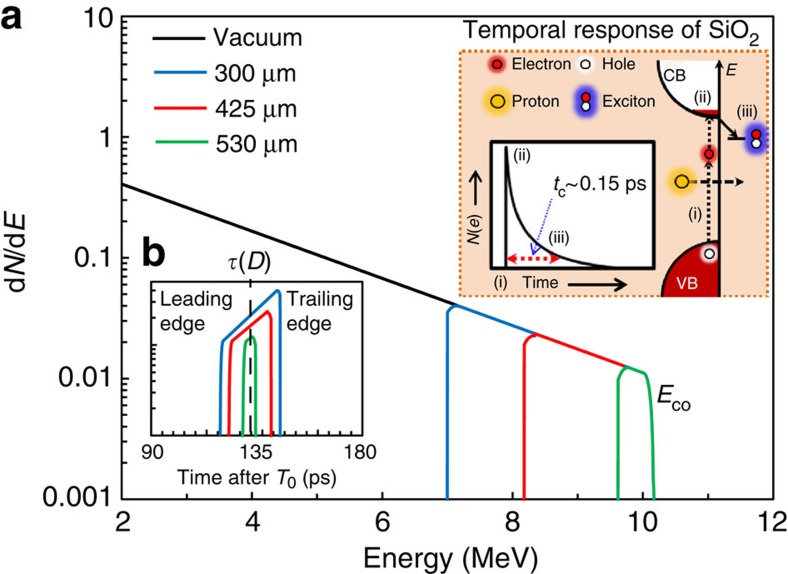
Ultrafast proton pulse metrology in SiO_2_. The dashed inset shows a schematic of the intraband dynamics and temporal response of an excited electron in *a*-SiO_2_. VB and CB are the valance and conduction bands, respectively, and *E* is the energy. Exciton formation provides the ultrafast de-excitation pathway for electrons in the CB^8^. The time constant for exciton formation is ∼0.15 ps (refs 8, 20). (**a**) Schematic of a TNSA proton spectrum (black trace) represented by a truncated exponential, with a proton temperature *T*=2 MeV and a high-energy spectral cutoff *E*_co_=10 MeV. The coloured traces show the bandwidth of the original spectrum that remains after proton stopping at different depths *D*, in bulk SiO_2_ (density 2.66 g cm^−3^) from stopping and range of ions in matter (SRIM) calculations^24^ (see Methods for more details). This returns a continuously decreasing bandwidth with respect to *D* (up to the stopping depth for *E*_co_, here ∼570 μm). The proton pulse duration *τ*(*D*) (**b**) is then simply the difference in time of flight for vacuum propagation (chosen here to be 5 mm) plus *D* for both the leading and trailing (high and low kinetic energy) edges of the remaining proton bandwidth. This illustrative schematic shows only the cumulative bandwidth narrowing of the originally broadband spectrum to reveal how short pulses can in principle be retrieved. It does not account for other aspects of the TNSA mechanism, such as energy-dependent cone narrowing^12^,^13^,^14^ critical for determination of the detailed proton pulse profile for a particular *D*.

To date the duration of laser-accelerated proton bunches has only been inferred from proton radiography^17^,^18^. For ultrafast applications, however, a more direct method of pulse metrology is desirable. In this section we show that the absolute measurement of laser-driven ion pulses is made possible by studying ultrafast ionization dynamics^8^,^9^,^20^ in ion-irradiated SiO_2_ (Fig. 1). We also demonstrate the application of this method to novel studies of the lifetime of excited conduction band electrons in proton-irradiated matter.

First we examine the temporal characteristics of protons accelerated via the target normal sheath acceleration (TNSA) mechanism (see Methods), although the technique described below is also applicable to ion bursts from alternative laser-driven acceleration schemes^14^,^23^. TNSA is an inherently broadband generation mechanism, producing a Maxwellian spectrum with a sharp, high-energy cutoff (*E*_co_). For nonrelativistic proton energies (MeV scale) this large energy spread causes the initially short ion pulse to stretch rapidly due to velocity dispersion, as it drifts from the source. The initial short pulse duration is still, however, preserved in narrow energy slices (bandwidths) of the spectrum. One way to recover this bandwidth is by stopping^24^ the lower-energy protons in bulk SiO_2_ (Fig. 1). The pulse duration *τ*(*D*) can then simply be estimated from time-of-flight considerations for the fastest and slowest edges of the bandwidth remaining at a given depth in the SiO_2_ (Fig. 1b). See Methods for further details on bandwidth narrowing and the corresponding pulse duration.

To measure *τ*(*D*) experimentally we study the lifetime of electrons excited into the conduction band of SiO_2_ by proton stopping. The schematic in the inset of Fig. 1 (dashed box) shows how this can provide the basis for an ultrafast detector. Prompt (<10^−15^ s) ionization by protons (i) results in the excitation of electrons into the conduction band (ii), where the Roman numerals correspond to labels in the inset of Fig. 1. Previous experiments based on uniform photoexcitation in SiO_2_ demonstrate a decay time constant of ∼0.15 ps for moderate excitation densities (∼10^19^ cm^−3^)^8^,^20^,^25^, with recombination being driven by ultrafast defect formation (iii) in the material^8^,^9^,^20^. Before recombination, however, these excited electrons are available to take part in free-free absorption of optical radiation (∼1 eV). It is this rapid switching in the optical transmission properties of irradiated SiO_2_ (transient opacity) that provides the platform for ultrafast metrology of proton pulses.

### Observation of the proton pulse duration

**Figure 2:**
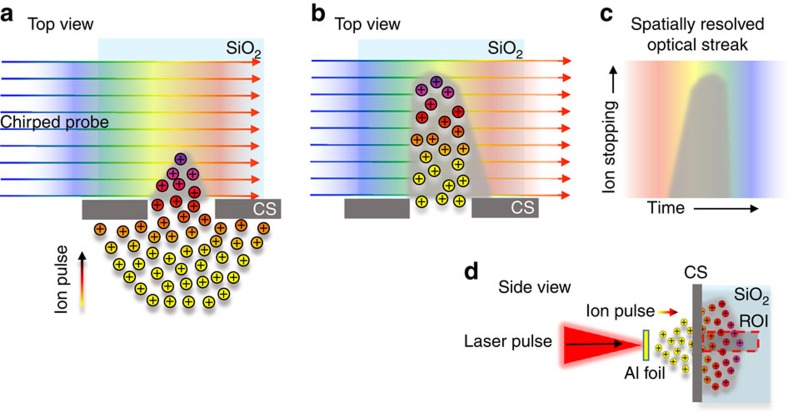
Schematic of the optical streaking technique for laser-driven proton bursts. Protons accelerated from a thin contamination layer on the rear surface of 10-μm-thick Au foils via the TNSA mechanism undergo electronic stopping in a high-purity SiO_2_ sample at ∼300 K placed 5 mm behind the interaction. The corresponding transient opacity (grey) is recorded using a synchronized 1,053-nm probe pulse with a variable linear frequency sweep, or chirp^26^. This permits observation of the interaction over time windows ranging from 0.4±0.02 (fully compressed pulse for direct imaging of interaction and represents the fundamental temporal resolution of this system) to ∼1,400 ps (maximum chirp for optical streaking). In **a** and **b** the proton bunch is incident from below and collimated to a width of 100 μm using a 1-mm-thick Al slit collimating slit (CS). Co-propagating keV electrons are stopped with the use of a 50-μm Al foil at the interaction facing surface of the SiO_2_ (not shown). A chirped pulse (>50 ps) is incident from the left. Different frequency components traverse the irradiated region at different times, thus encoding the temporal evolution in the observed spectrum. It is important to note that the bandwidth of the optical probe (4 nm) is narrow compared with the width of the absorption spectrum for conduction band electrons in SiO_2_. The optical streak is obtained by spectrally resolving the chirped pulse (**c**) using a 1-m imaging spectrometer with a 1,200 l mm^−1^ grating (dimensions 10 × 10 cm). The region of interest (ROI, (**d**)) for the ion burst interaction is a 10-μm scale slice along the central axis of the driving laser pulse. This is imaged onto the entrance slit of the spectrometer with a magnification of ∼10. The fundamental temporal resolution of the system described here is limited only by the resolution of the spectrometer^26^. For a 200-ps probe this system provides a resolution of 0.45±0.05 ps.

**Figure 3:**
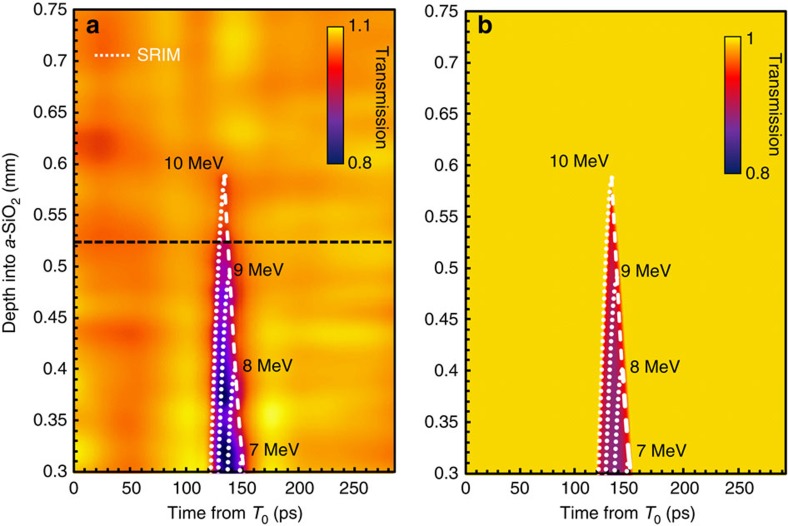
Optical streaking of transient ion-induced opacity in SiO_2_. (**a**) An experimentally obtained optical streak for opacity induced in SiO_2_ by the stopping of a TNSA proton pulse with a spectral endpoint energy of 10±0.5 MeV generated by the TARANIS laser. The data are normalized to the average full transmission The on laser axis proton flux in the SiO_2_ ranges from 250±50 μm^−2^ for *D*=300 μm to 100±50 μm^−2^ for *D*=530 μm as inferred from radiochromic film measurements. The average excited electron density is measured to be <10^19^ cm^−2^ from interferometry of the interaction region using a fully compressed probe pulse and Wollaston prism. In **a** and **b** the colour scale goes from full transmission (light) to 80% transmission (0.8, dark). There is a slight modulation due to noise on the experimentally obtained streak. The data are shown for depths >300 μm due to small bevelled edge (∼100 μm) on the SiO_2_ sample increasing uncertainty in the measured transmitted light. It is important to note however that the signal due to ions (starting at ∼120 ps) is not compromised by earlier opacity from prompt electrons/X-rays at this depth. The white dotted lines represent stopping and range of ions in matter (SRIM)^24^ calculations for monoenergetic protons (labelled) stopping in SiO_2_. The white dashed line corresponds to the 1/*e* level of the peak in the stopping curve at the end of range for these calculations. (**b**) Modelling for the expected temporal evolution of opacity in SiO_2_ for the proton spectrum. This modelling takes into account the energy-dependent cone angle of the emission as measured using radiochromic film stacks^14^. The key assumptions made for **b** are that the opacity generated is linear with stopping power^24^ and that the response time of the detector is 0.45 ps.

**Figure 4:**
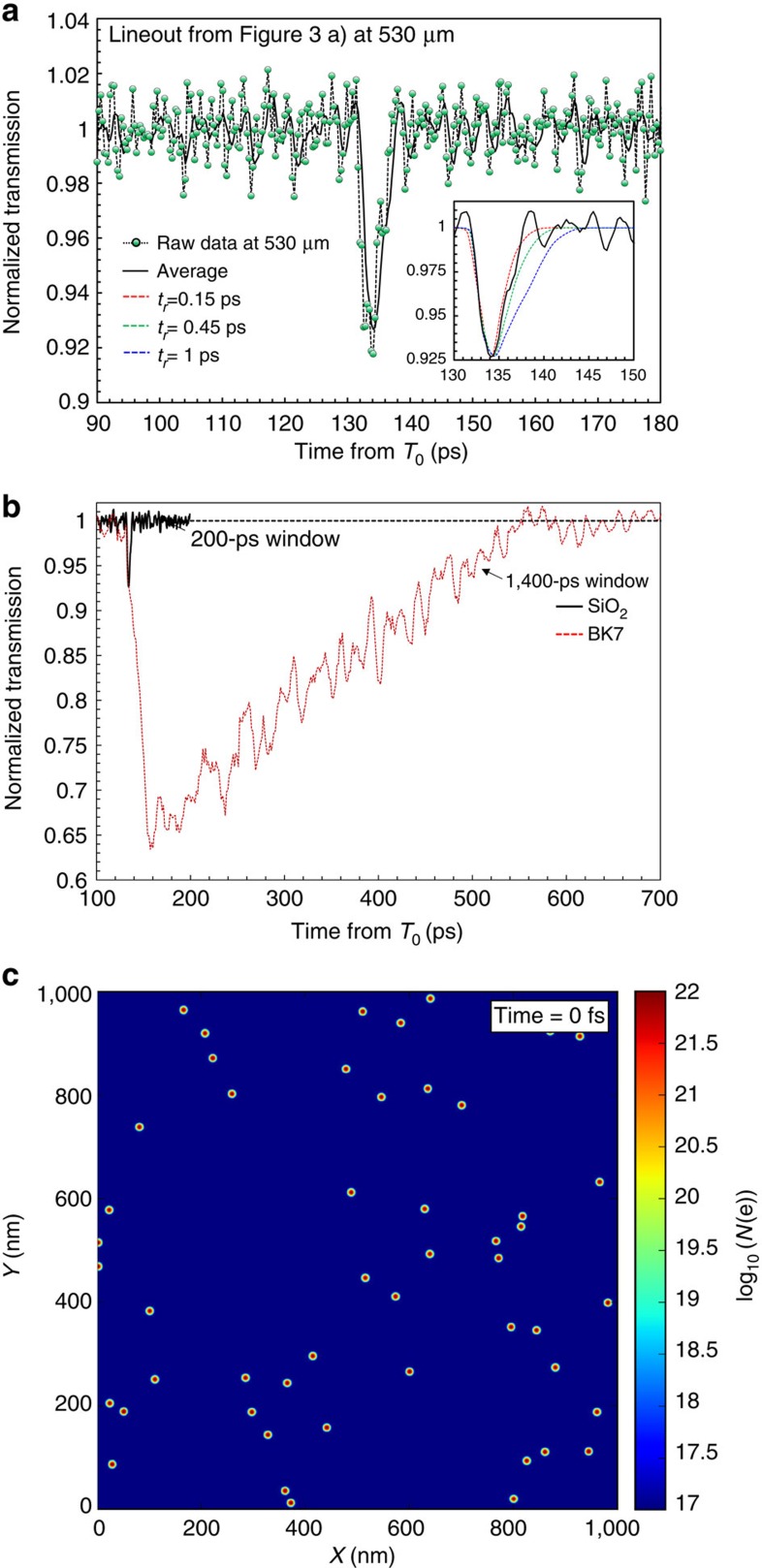
Ultrafast metrology of proton bursts and their interactions in condensed matter. (**a**) Background corrected data (points) and moving average signal over seven adjacent points (solid black line) for the transient opacity at a depth of 530 μm in optical streak presented in Fig. 3a. All times are shown with respect to the interaction time, *T*_0_. The observed duration of the transient opacity is 3.5±0.7 ps. The inset of **a** shows a comparison of the average signal level with modelling of the optical streaking adjusted for different detector response times, *t*_r_. The best fit for the observed temporal profile is found to correspond to *t*_r_=0.45 ps, which is close to the resolution limit of the optical pulse^26^,^27^. (**b**) A direct comparison between the response time for TNSA pulse interactions in SiO_2_ (black line, as in **a** and BK7 (red dotted line) at depths of 530 μm and 610 μm to account for the slightly different densities of the two media—2.66 and 2.31 g cm^−3^, respectively. The horizontal dashed line represents the mean of full transmission. (**c**) The result of Monte–Carlo-based modelling for the instantaneous dose distribution at a depth of 530 μm for a proton flux of 50 μm^−2^. The colour scale is in log of free electron density (*N*(e)).

Figure 2 shows a schematic for the optical streaking^26^ technique used to temporally resolve the transient opacity in SiO_2_ induced by laser-driven ion pulses. To experimentally verify this technique the TARANIS laser facility at Queens University Belfast^27^ is used to accelerate protons via the TNSA mechanism to a spectral cutoff energy (*E*_co_) of 10±0.5 MeV. This pulse then interacts with a high-purity SiO_2_ sample placed 5±0.5 mm behind the generation point. A typical optical streak obtained from this setup is shown in Fig. 3a. In agreement with full modelling for the optical streak (based on Monte Carlo simulations, Fig. 3b), the duration of the transient opacity gets successively shorter with respect to *D* due to bandwidth narrowing as a result of low-energy proton stopping. For *D*=530±10 μm the duration of the opacity is measured to be 3.5±0.7 ps (Fig. 4a). This is in excellent agreement with the expected proton pulse duration *τ*(*D*) of ∼3.3 ps from both calculations (green trace, Fig. 1b) and modelling (Fig. 3b).

In fact, using this modelling of the optical streak we can analyse the temporal profile of the measured proton pulse in some detail. The resolution of this measurement is 0.45±0.05 ps, which corresponds to a proton pulse bandwidth of <0.1 MeV for the conditions described in Fig. 4a. We find that best agreement between experiment and modelling is obtained for an assumed detector response time *t*_r_ of 0.45 ps (green dashed trace, inset, Fig. 4a). This places an upper bound of 0.1 MeV on the decay constant of the TNSA spectral intensity above *E*_co_ and an upper limit of <0.2 ps on the lifetime of excited electrons in the conduction band of the irradiated sample SiO_2_ (from temporal profile fitting, inset, Fig. 4a). Furthermore, this analysis demonstrates that proton straggling does not lead to a significant increase in the observed pulse duration beyond what is expected due to slowing in SiO_2_. In all, this analysis confirms the efficacy of SiO_2_ as a sub-picosecond resolution detector for proton bursts. Finally, our ultrafast metrology verifies that there is negligible thermal spread imposed on the nascent bunch during the acceleration mechanism for the fastest protons (>6 MeV), that is, the protons are accelerated from an initially cold cathode for our experimental conditions. The observation of emission from a cold cathode is in agreement with previous experimental measurements of the transverse emittance of the TNSA beam on similar specification systems^28^.

It is worth noting that for proton bunches with an inherently narrow energy spread, stopping in bulk SiO_2_ is not required to achieve ultrafast temporal resolution. In this scenario the bulk sample can be replaced by a thin SiO_2_ pellicle and the proton pulse duration can be obtained using a near-collinear probing geometry^20^. For proton beams with sufficiently high kinetic energy this will have a negligible effect on beam quality and is a possible route to online temporal metrology. In addition, this near-collinear geometry can provide information on the two-dimensional beam spatial/angular distribution by imaging the opacity induced by the proton–SiO_2_ interaction directly onto a charge-coupled device as an ultrafast replacement for comparatively slow scintillation screens.

### Ultrafast ion damage in matter

Next we demonstrate how this technique can be applied more generally to study ultrafast dynamics in proton-irradiated condensed matter. In Fig. 4b we show a direct comparison of the lifetime of conduction band electrons in SiO_2_ and borosilicate glass (BK7) for identical proton pulse conditions to those presented in Fig. 3a. BK7 is a multicomponent derivative of SiO_2_, representing a low-cost alternative for laboratories due to its similar thermal shock and optical properties. However, as can be seen, the proton-induced transient opacity is observed to have a >400-ps recovery time to full transmission (red dotted trace, Fig. 4b). Photoexcitation experiments in BK7 suggest that this long recovery can be interpreted as being due to occupied interstitial levels preventing rapid relaxation of the hot electron population^29^. However, those experiments also indicate that the lifetime of photoexcited electrons is ∼5,000 ps. Our measurement of 430±20 ps, while being ∼100 times longer than that of SiO_2_, is significantly shorter than 5,000 ps.

## Discussion

One hypothesis for this discrepancy lies in the nature of proton interactions in condensed matter. Monte Carlo modelling for damage tracks in SiO_2_ shows that an instantaneous proton flux (that is, time=0 fs) of 50 μm^−2^ produces a strongly inhomogeneous dose distribution with excited electron densities falling from >10^21^ cm^−3^ to equilibrium level over transverse widths <4 nm (Fig. 4c). From simple electronic diffusivity considerations this distribution will evolve rapidly over ps timescales in comparison with homogenous photoexcitation^3^. While a detailed study of this evolution is beyond the scope of the work presented here, the observation of significantly reduced recovery timescales in BK7 provides an example of the new insights into physical processes this metrology can provide for transient dynamics resulting from proton–matter interactions. Furthermore, the high degree of synchronicity between the proton and probe sources means that our technique can be readily extended to study the evolution of specific transient defects by changing the probe wavelength, either through frequency conversion in crystals or nonlinear conversion using either gas or relativistic plasma^21^,^22^ sources, to temporally resolve absorption bands for specific excited species.

Looking to the future, the prospect of generating narrow-bandwidth proton spectra with femtosecond duration driving lasers^14^ offers the possibility of extending this technique to timescales suitable for investigating primary radiation events during the rising edge of the interaction using femtosecond scale probing. Another possibility is the potential to extend work examining energy transfer in non-equilibrium warm dense matter generated through isochoric heating using laser-accelerated protons^30^. For the present TNSA source, schemes that require the maintenance of high proton beam quality can be realized using a simple magnetic spectrometer and slit arrangement, or more complicated chromatic focusing schemes using a laser-driven electrostatic lens^31^ to select the desired flux and energy bandwidth (or pulse duration) instead of via stopping in bulk material. This will permit ultrafast studies of proton implantation and resulting damage at well-specified depths in materials.

## Methods

### Target normal sheath acceleration

During intense laser–foil (μm scale thickness) interactions an accelerating potential at the rear surface of the foil is rapidly established by electrons driven through the target from the front surface by the intense laser field. As they exit the foil, these electrons ionize a thin hydrocarbon contamination layer on the rear surface leaving a net positive charge in their wake. Simulations show that this process leads to the formation of a charge separation sheath producing a strong electrostatic field exceeding 1 TV m^−1^, which provides a transient accelerating potential for the rear-surface ions^14^. However, since the fastest electrons only contribute to this sheath for a fraction of the driving laser duration, the peak in this accelerating potential is very short-lived. It is this ultrafast evolution of the accelerating potential that provides the basis for ultrashort ion pulse generation during TNSA.

### Stopping of protons in SiO_2_ for TNSA bandwidth/pulse duration selection

As the broad bandwidth of TNSA protons transverses the SiO_2_ sample, they continuously lose kinetic energy, that is, a 10-MeV proton in vacuum has <<1 eV kinetic energy after ∼570 μm of SiO_2_. This implies that the bandwidth of the TNSA spectrum continually narrows and shifts towards lower energies with respect to *D*. At a given depth the remaining bandwidth is bounded on the leading edge by the fastest protons. These protons have a well-defined energy (*E*_co_ before entering the SiO_2_). Next the trailing/slowest edge is bounded by the lowest-energy protons that have yet to be stopped at that depth^24^. Together these edges define a sharply bounded, depth-dependent bandwidth that narrows as the fastest protons slow to thermal energies (which implies that all protons with lower initial kinetic energy are already stopped). This is illustrated in Fig. 1b where the pulse duration (obtained from the bandwidth) is shown to reduce with respect to *D*.

## Additional information

**How to cite this article:** Dromey, B. *et al*. Picosecond metrology of laser-driven proton bursts. *Nat. Commun.* 7:10642 doi: 10.1038/ncomms10642 (2016).

## References

[b1] Baldacchino G. Pulse radiolysis in water with heavy-ion beams. A short review. Rad. Phys. Chem. 77, 1218–1223 (2008).

[b2] Murat M., Akkerman A. & Barak J. Spatial distribution of electron-hole pairs induced by electrons and protons in SiO_2_. IEEE Trans. Nucl. Sci. 51, 3211–3218 (2004).

[b3] Osmani O., Medvedev N., Schleberger M. & Rethfeld B. Energy dissipation in dielectrics after swift heavy-ion impact: a hybrid model. Phys. Rev. B 84, 214105 (2011).

[b4] Medvedev N. . Early stage of the electron kinetics in swift heavy ion tracks in dielectrics. Phys. Rev. B 82, 125425 (2010).

[b5] Schiwietz G., Czerski K., Roth M., Staufenbiel F. & Grande P. L. Femtosecond dynamics—snapshots of the early ion-track evolution. Nucl. Instrum. Methods Phys. Res. B 225, 4–26 (2004).

[b6] Kluth P. . Fine structure in swift heavy ion tracks in amorphous SiO_2_. Phys. Rev. Lett. 101, 175503 (2008). 18999762 10.1103/PhysRevLett.101.175503

[b7] Chipman D. M. Absorption spectrum of OH radical in water. J. Phys. Chem. A 112, 13372–13381 (2008). 19053573 10.1021/jp807399b

[b8] Guizard J. . Time resolved study of colour centre formation SiO_2_. J. Phys. Condens. Matter 8, 1281–1290 (1996).

[b9] Hosono H., Kawazoe H. & Matsunami N. Experimental evidence for frenkel defect formation in amorphous SiO2 by electronic excitation. Phys. Rev. Lett. 80, 317–320 (1998).

[b10] Rigaud O. . Exploring ultrashort high-energy electron-induced damage in human carcinoma cells. Cell Death Dis. 1, e73 (2010). 21364677 10.1038/cddis.2010.46 PMC3032345

[b11] Baudrenghien P. & Mastoridis T. Longitudinal emittance blowup in the large hadron collider. Nucl. Instrum. Methods Phys. Res. A 726, 181–190 (2013).

[b12] Clark E. L. . Energetic heavy-ion and proton generation from ultra-intense laser-plasma interactions with solids. Phys. Rev. Lett. 85, 1654–1657 (2000). 10970581 10.1103/PhysRevLett.85.1654

[b13] Snavely R. A. . Intense high-energy proton beams from petawatt-laser irradiation of solids. Phys. Rev. Lett. 85, 2945–2948 (2000). 11005974 10.1103/PhysRevLett.85.2945

[b14] Macchi A., Borghesi M. & Passoni M. Ion acceleration by superintense laser-plasma interaction. Rev. Mod. Phys. 85, 751–793 (2013).

[b15] Yogo A. Application of laser-accelerated protons to the demonstration of DNA double-strand breaks in human cancer cells. Appl. Phys. Lett. 94, 181502 (2009).

[b16] Pelka A. . Ultrafast melting of carbon induced by intense proton beams. Phys. Rev. Lett. 105, 265701 (2010). 21231678 10.1103/PhysRevLett.105.265701

[b17] Borghesi M. . Electric field detection in laser-plasma interaction experiments via the proton imaging technique. Phys. Plasmas 9, 2214–2220 (2002).

[b18] Abicht F. . Tracing ultrafast dynamics of strong fields at plasma-vacuum interfaces with longitudinal proton probing. Appl. Phys. Lett. 105, 034101 (2014).

[b19] Correa A. A. . Nonadiabatic forces in ion-solid interactions: the initial stages of radiation damage. Phys. Rev. Lett. 108, 213201 (2012). 23003250 10.1103/PhysRevLett.108.213201

[b20] Audebert P. . Space–time observation of an electron gas in SiO2. Phys. Rev. Lett. 73, 1990–1993 (1994). 10056940 10.1103/PhysRevLett.73.1990

[b21] Kiefer D. . Relativistic electron mirrors from nanoscale foils for coherent frequency upshift to the extreme ultraviolet. Nat. Commun. 4, 1763 (2013). 23612304 10.1038/ncomms2775 PMC3644103

[b22] Dromey B. . Coherent synchrotron emission from electron nanobunches formed in relativistic laser-plasma interactions. Nat. Phys. 8, 804–808 (2012).

[b23] Jung D. . Scaling of ion energies in the relativistic-induced transparency regime. Laser Part. Beams 33, 695–703 (2015).

[b24] Ziegler J. F., Ziegler M. D. & Biersack J. P. SRIM—the stopping and range of ions in matter. Nucl. Instrum. Methods B 268, 1027–1036 (2010).

[b25] Riedel R. . Single-shot pulse duration monitor for extreme ultraviolet and X-ray free-electron lasers. Nat. Commun. 4, 1731 (2013). 23591898 10.1038/ncomms2754

[b26] Polli D., Brida D., Mukamel S., Lanzani G. & Cerullo G. Effective temporal resolution in pump-probe spectroscopy with strongly chirped pulses. Phys. Rev. B 82, 053809 (2010).

[b27] Dzelzainis T. . The TARANIS laser: a multi-Terawatt system for laser-plasma investigations. Laser Part. Beams 28, 451–461 (2010).

[b28] Cowan T. E. . Ultralow emittance, multi-MeV proton beams from a laser virtual-cathode plasma accelerator. Phys. Rev. Lett. 92, 204801 (2004). 15169357 10.1103/PhysRevLett.92.204801

[b29] Horn A., Kreutz E. W. & Poprawe R. Ultrafast time-resolved photography of femtosecond laser induced modifications in BK7 glass and fused silica. Appl. Phys. A 79, 923–925 (2004).

[b30] White T. G. . Observation of inhibited electron-ion coupling in strongly heated graphite. Sci. Rep. 2, 889 (2012). 23189238 10.1038/srep00889 PMC3506979

[b31] Kar S. . Dynamic control of laser-produced proton beams. Phys. Rev. Lett. 100, 105004 (2008). 18352198 10.1103/PhysRevLett.100.105004

